# De Novo Autosomal Dominant Cutis Laxa Type 3 With Global Developmental Delay and Musculoskeletal Features of Refractory Rickets

**DOI:** 10.1002/ccr3.70105

**Published:** 2025-02-26

**Authors:** Subhangi Chandan, Jay Gohri, Arshia Jolly, Mayuri Chaurasia

**Affiliations:** ^1^ Department of Pediatrics Lady Hardinge Medical College Delhi India; ^2^ Department of Medicine JSS Medical College Mysore India; ^3^ Department of Medical Genetics KMC Mangalore Mangalore Karnataka India; ^4^ Department of Pediatrics NIMS Jaipur India

**Keywords:** developmental delay, genetics, neurology, pediatrics

## Abstract

Cutis laxa is a genetically heterogeneous disorder characterized primarily by loose, redundant skin with abnormal wrinkling and elasticity. It is an exceptionally rare condition, with an estimated prevalence of < 1 in 1,000,000 individuals. In addition to the distinctive cutaneous manifestations, cutis laxa can present with a constellation of other features, including progeroid appearance, growth retardation, and developmental delays. We report a case of a 26‐month‐old girl who presented with features similar to nutritional rickets with global developmental delay and some additional features of joint and skin hyper‐laxity in the backdrop of severely low vitamin D levels. The patient, however, failed to respond to the conventional treatment for rickets. Subsequent genetic testing revealed an autosomal dominant form of cutis laxa caused by an exceedingly rare c.377G>A (p.Arg126His) substitution in the ALDH18A1 gene, which encodes the bifunctional enzyme catalyzing the final steps of de novo phospholipid biosynthesis. The present case highlights the diagnostic challenges posed by cutis laxa, as the clinical manifestations can overlap with other conditions, leading to potential misdiagnosis or delayed recognition. The rarity of this disorder, combined with its phenotypic variability, underscores the importance of raising awareness among clinicians and expanding the literature to encompass the full spectrum of presentations associated with cutis laxa.

**Summary**

- This case report highlights a rare and underreported presentation of autosomal dominant cutis laxa type 3 (ADCL3), expanding our understanding of its phenotypic spectrum and emphasizing the importance of considering genetic disorders in atypical presentations of common conditions.
- The overlapping musculoskeletal features with rickets create a significant diagnostic challenge, especially given the limited literature on cutis laxa. This underscores the need for a high index of suspicion and the value of genetic testing in resolving complex clinical presentations, particularly in regions where nutritional deficiencies are prevalent.

## 1 Introduction

Cutis laxa (CL) is a rare genetic disorder associated with skin hypoelasticity and looseness with or without the presence of systemic features depending on the type of disease. Both acquired and inherited forms of the disease have been documented, despite the paucity that exists in the available literature for this rare case. The heritable forms are further divided into three based on mode of inheritance: AD, AR, and XLR [1].

Autosomal dominant CL is known to show clinical variability with the presence of benign visceral manifestations and genetic heterogeneity with clinical variability. We do not expect the autosomal dominant variant of CL to impact life expectancy negatively. Multiple types of ADCL have been described based on the gene involved like elastin (type 1), fibulin 5 (type 2), and ALDH18A1 (type 3). De novo mutations have been found in the aldehyde dehydrogenase 18 family member A1 (ALDH18A1), which are extremely rare and have been reported in fewer than 20 cases worldwide [2].

From a clinical point of view, this particular mutation is associated with progeroid features and neurological abnormalities that may not always be present in the patient and is only relatively diagnosed by molecular studies. As ALDH18A1, which encodes for delta1‐pyrroline‐5‐carboxylate synthase (P5CS), is found to be responsible for this condition and a missense mutation (more commonly), it has been linked to symptoms like worsening nerve damage, cataracts, loose skin, joint problems, and metabolic issues; all of which can be found in ADCL type 3 [3].

Since there is no proper treatment available for this condition, the patient is managed with regular follow‐ups and counseling.

## 2 Case History

A 26‐month‐old female patient presented at the genetics outpatient clinic for follow‐up evaluation of gross motor developmental delay since approximately 12 months of age. Prior to 1 year of age, the patient's development was unremarkable. However, upon reaching 12 months, her parents expressed concerns regarding delayed developmental milestones to the pediatrics outpatient department. The patient achieved head control at 8–9 months and independent sitting at approximately 12 months of age. Initially, the patient was treated for suspected refractory rickets, but upon lack of clinical improvement, she was referred to the genetics clinic for further diagnostic evaluation. The patient's medical history was negative for any prenatal, perinatal, or postnatal events, seizures, hypoxic–ischemic encephalopathy, rashes, or joint pain.

The patient was born via normal vaginal delivery to non‐consanguineous parents, with a 25‐year‐old primigravida mother, following an uneventful antenatal period. Her birth weight was 2.8 kg, length 58 cm, and head circumference 32 cm. The patient was exclusively breastfed until 7 months of age and currently maintains a normal diet. While exhibiting appropriate fine motor and social skill development, the patient demonstrated profound gross motor delays, with a developmental quotient of 62. Acquisition of head control occurred at 9 months, independent sitting at 14 months, and supported standing and ambulation at 24 months. Additionally, the patient exhibited mild language delays, presenting with jargon speech upon evaluation.

**FIGURE 1:**
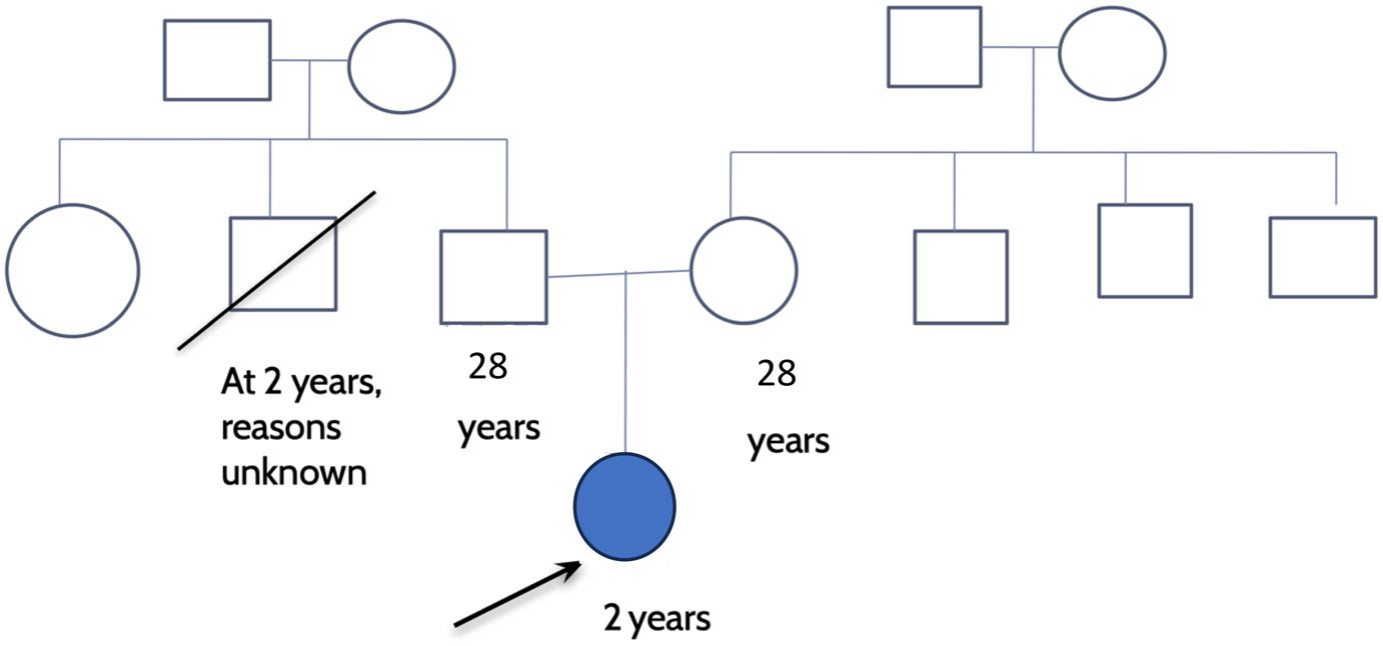
Pedigree analysis of the patient.

The family history was unremarkable for any known genetic abnormalities or developmental delays (Figure 1). The family belongs to a lower socioeconomic stratum.

## 3 Methodology

**FIGURE 2:**
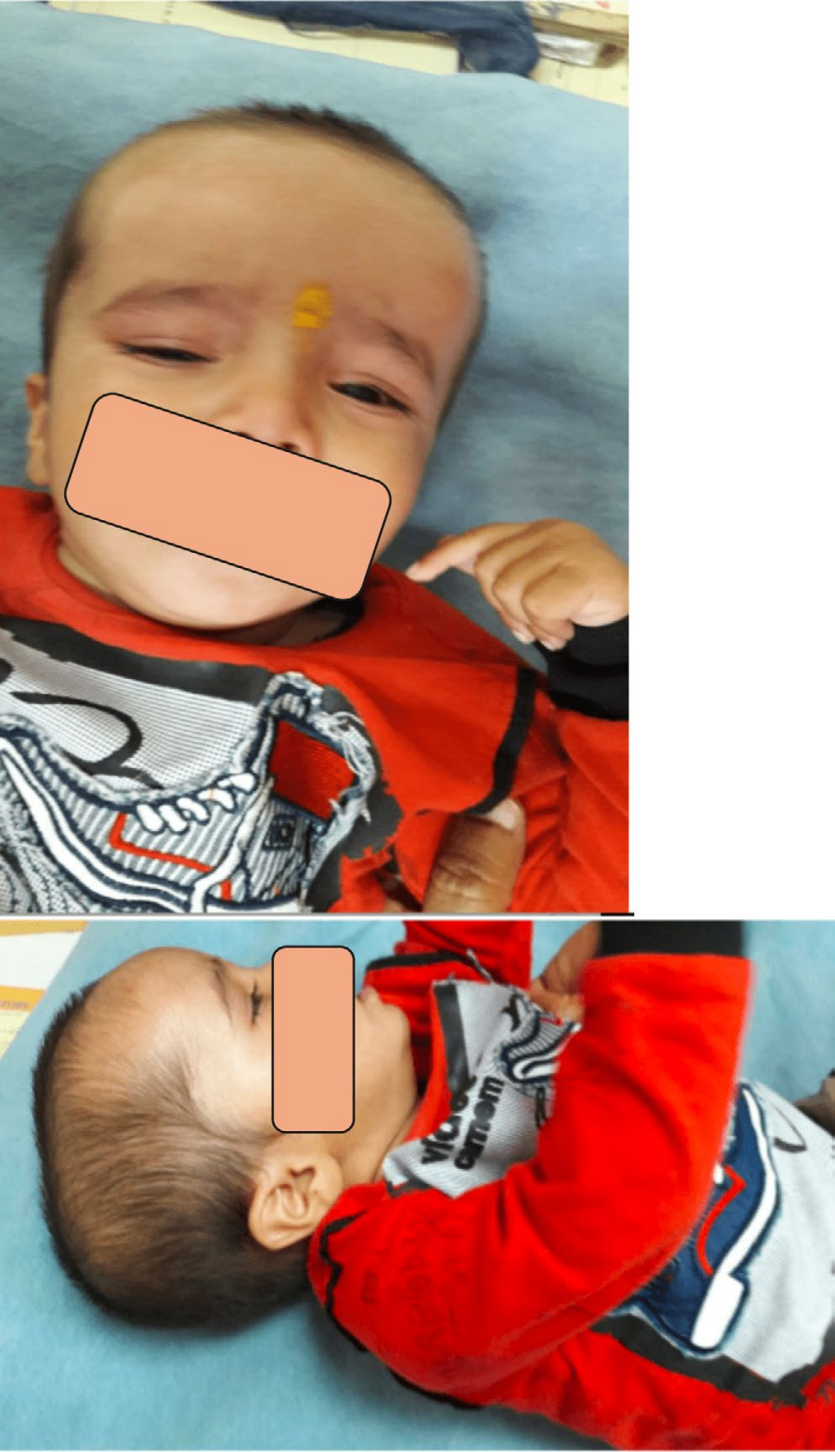
(A and B) Frontal bossing noted in the child.

**FIGURE 3:**
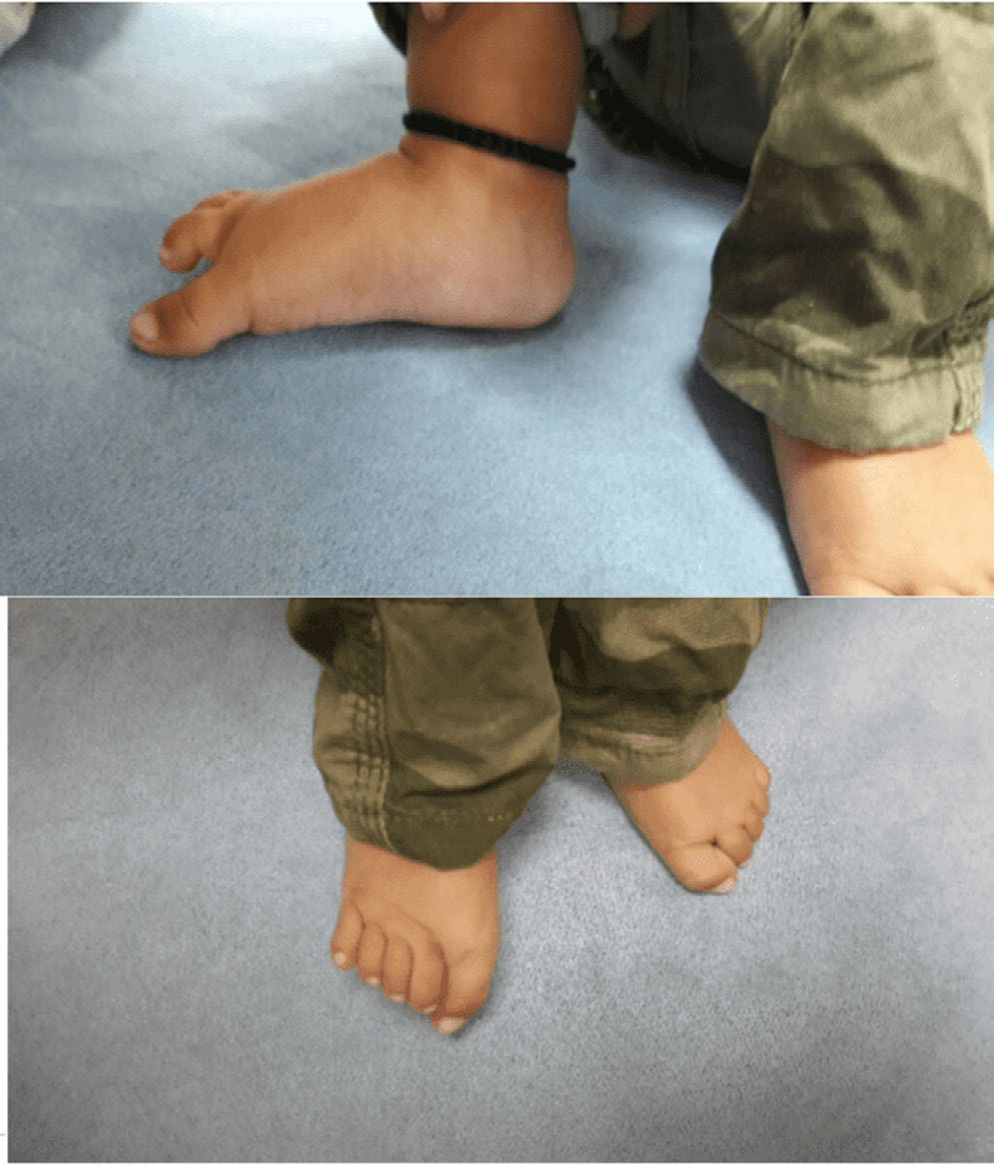
(A and B) Pes planus and genu recurvatum.

Upon physical examination, the patient was calm and nonirritable. Her anthropometric measurements were as follows: weight 11 kg (< 3rd percentile), length 78 cm (< 3rd percentile), and head circumference 40 cm (< −3 standard deviations). Vital signs were stable, with a pulse rate of 60 beats per minute, blood pressure of 90/50 mmHg, and temperature of 98.2°F (36.8°C). No pallor, lymphadenopathy, cyanosis, or icterus was noted. A general head‐to‐toe examination revealed a prominent broad forehead with associated frontal bossing (Figure 2). All fontanelles were closed, and no gross facial dysmorphology was observed. The neck and upper limbs appeared normal. The lower limbs exhibited pes planus and genu recurvatum (Figure 3).

Neurological, cardiovascular, and respiratory system examinations were unremarkable.

Musculoskeletal examination revealed moderate skin hyperextensibility with hypermobile joints, but no associated hypotonia. Brain magnetic resonance imaging (MRI) findings were normal.

Laboratory investigations showed a serum calcium level of 9 mEq/L, serum phosphate level of 2.0 mEq/L, and alkaline phosphatase (ALP) level of 380 IU/L. The patient was initially treated as a case of nutritional rickets for several months with multiple doses of oral and intramuscular vitamin D (nadir 14.6 U), with vitamin D levels reaching 280.5 U. However, no significant improvement in the patient's symptoms was observed, even with concomitant calcium supplementation. Subsequently, the patient was referred to a geneticist for further workup of possible refractory rickets.

## 4 Results

Whole‐exome sequencing was performed, revealing a heterozygous c.377G>A missense mutation (substitution of arginine by histidine at position 126) in exon 15 of the ALDH18A1 gene on chromosome 10. The patient was diagnosed with autosomal dominant cutis laxa with a mutation in the ALDH18A1 gene (de novo), also known as cutis laxa, autosomal dominant, type 3 (ADCL3). The parents were counseled accordingly, and the patient was scheduled for regular follow‐up to monitor for potential future manifestations of this condition.

## 5 Discussion

De novo mutation of ALDH18A1 associated with ADCL type 3 has been found to be the mildest subtype of cutis laxa with mostly skin and joint findings and hardly any associated pulmonary or cardiovascular comorbidities, as are seen with type 1 and type 2 ADCL.

The initial misdiagnosis of nutritional rickets in our patient emphasizes the importance of maintaining a broad differential diagnosis, especially in cases with atypical or refractory presentations. The combination of facial and foot deformities, coupled with low vitamin D levels, initially led to a presumptive diagnosis of rickets, a common condition in economically disadvantaged populations in India. However, the lack of response to conventional treatment prompted further investigation, ultimately leading to the molecular diagnosis of ADCL3 through whole‐exome sequencing [2].

Our case contributes to the growing body of knowledge on ADCL3 by demonstrating a milder phenotype associated with the p.Arg126His mutation. This presentation aligns more closely with cases involving mutations in exon 15 of the ALDH18A1 gene, such as the cG1867A mutation reported by Kapoor et al. The proximity of the p.Arg126His substitution to the more common p.Arg138 substitutions on the PSC5 domain may explain some similarities in presentation, while also accounting for the observed phenotypic differences. Our patient's clinical presentation differed significantly from the previously reported case, lacking progeroid features, truncal hypotonia, or triangular facies. This variability in presentation underscores the phenotypic spectrum of ADCL3 and highlights the challenges in its diagnosis [4].

Early molecular diagnosis is crucial for ADCL3, as it enables appropriate genetic counseling and facilitates anticipatory management of potential complications. While no curative treatments currently exist, supportive care and management of specific manifestations can significantly improve quality of life for affected individuals. Regular follow‐ups, including ophthalmological evaluations, echocardiography, and neuroimaging, are essential, as is ruling out potential differentials such as pseudoxanthoma elasticum and Ehlers–Danlos syndrome [5].

## 6 Conclusion

We aim to highlight this particularly rare mutation of cutis laxa with p.Arg126His substitution, which has only one other reported case but with a completely different clinical presentation. Our patient's clinical features were much milder with minimal comorbidities compared to previously described cases in the available literature. This case emphasizes the importance of considering genetic testing in cases of refractory rickets, especially in the context of vitamin D deficiency. It also expands our understanding of the phenotypic spectrum of ADCL3, highlighting the need for a high index of suspicion in atypical presentations of common nutritional disorders. Future research should focus on further delineating the genotype–phenotype correlations in ADCL3 to improve diagnosis and management of this rare genetic condition.

## Author Contributions

**Subhangi Chandan:** conceptualization, data curation, investigation, methodology, project administration, supervision, validation, visualization, writing – original draft, writing – review and editing. **Jay Gohri:** investigation, project administration, validation, visualization, writing – original draft, writing – review and editing. **Arshia Jolly:** resources, software, supervision, validation, visualization, writing – original draft, writing – review and editing. **Mayuri Chaurasia:** supervision, validation, visualization, writing – original draft.

## Ethics Statement

The authors have nothing to report.

## Consent

Written informed consent was obtained from the patient's parents/guardians to publish this report in accordance with the journal's patient consent policy.

## Conflicts of Interest

The authors declare no conflicts of interest.

## Data Availability

Data sharing is not applicable to this article as no new data were created or analyzed in this study.
